# Does group size matter during collaborative skills learning? A randomised study

**DOI:** 10.1111/medu.14791

**Published:** 2022-03-16

**Authors:** Laerke Marijke Noerholk, Anne Mette Morcke, Kulamakan Kulasegaram, Lone N. Nørgaard, Lotte Harmsen, Lisbeth Anita Andreasen, Nina Gros Pedersen, Vilma Johnsson, Anishan Vamadevan, Martin Grønnebæk Tolsgaard

**Affiliations:** ^1^ Copenhagen Academy for Medical Education and Simulation (CAMES) Copenhagen University Hospital ‐ Rigshospitalet Copenhagen Denmark; ^2^ Centre for Educational Development Aarhus University Aarhus Denmark; ^3^ Department of Family & Community Medicine University of Toronto Toronto Canada; ^4^ Center of Fetal Medicine, Department of Obstetrics Copenhagen University Hospital ‐ Rigshospitalet Copenhagen Denmark; ^5^ Department of Gynecology and Obstetrics Copenhagen University Hospital ‐ Hvidovre Copenhagen Denmark; ^6^ Fetal Medicine Unit, Department of Obstetrics and Gynaecology Copenhagen University Hospital ‐ Herlev Copenhagen Denmark; ^7^ Department of Clinical Medicine, Faculty of Health and Medical Sciences University of Copenhagen Copenhagen Denmark

## Abstract

**Background:**

Collaborative skills learning in the form of dyad learning compared with individual learning has been shown to lead to non‐inferior skills retention and transfer. However, we have limited knowledge on which learning activities improve collaborative skills training and how the number of collaborators may impact skills transfer. We explored the effects of skills training individually, in dyads, triads or tetrads on learning activities during training and on subsequent skills transfer.

**Methods:**

In a randomised, controlled study, participants completed a pre‐post‐transfer‐test set‐up in groups of one to four. Participants completed 2 hours of obstetric ultrasound training. In the dyad, triad and tetrad group participants took turns actively handling the ultrasound probe. All performances were rated by two blinded experts using the Objective Structured Assessment of Ultrasound Skills (OSAUS) scale and a Global Rating Scale (GRS). All training was video recorded, and learning activities were analysed using the Interactive‐Constructive‐Active‐Passive (ICAP) framework.

**Results:**

One hundred one participants completed the simulation‐based training, and ninety‐seven completed the transfer test. Performance scores improved significantly from pre‐ to post‐test for all groups (p < 0.001, ηp^2^ = 0.55). However, group size did not affect transfer test performance on OSAUS scores (p = 0.13, ηp^2^ = 0.06) or GRS scores (p = 0.23, ηp^2^ = 0.05). ICAP analyses of training activities showed that time spent on non‐learning and passive learning activities increased with group size (p < 0.001, ηp^2^ = 0.31), whereas time spent on constructive and interactive learning activities was constant between groups compared with singles (p < 0.001, ηp^2^ = 0.72).

**Conclusion:**

Collaborative skills learning in groups of up to four did not impair skills transfer despite less hands‐on time. This may be explained by a compensatory shift towards constructive and interactive learning activities that outweigh the effect of shorter hands‐on time.

## Abstract 2

Collaborative skills learning in group sizes of up to four participants is reported in this work to not impair skills transfer despite individual trainees having less hands‐on time.

## 1 INTRODUCTION

Globally, medical schools are responding to the need for more doctors by admitting a greater number of medical students and increasing class sizes. Unfortunately, this increased intake may have detrimental effects on students' learning given the finite resources available for clinical training.^1^, ^2^, ^3^ As a result, educators have called for more cost‐effective methods of training and instructional methods that rely less on faculty resources than the traditional one‐to‐one apprenticeship model, in particular for learning clinical skills. Currently, frontline research proposes an increased focus on the role of collaborative learning of skills in undergraduate medical education.^4^, ^5^, ^6^

Collaborative learning is defined as ‘groups of learners working together to solve a problem, complete a task or create a product’.^7^ Recent studies have shown that collaborative skills learning in the form of dyad learning (i.e. learning in pairs) compared with individual learning in a simulated setting leads to improved skills retention^4^, ^8^ and non‐inferior skills transfer to the clinical workspace.^9^ In addition, dyad learning is thought to be more cost‐effective than individual learning because it only requires half the instructional resources, while producing similar or better educational outcomes.^10^, ^11^ The positive effects of collaborative learning may be mediated through mechanisms such as peer observation,^8^ cognitive co‐construction of knowledge,^12^ reduced cognitive load,^5^ social interdependence^13^ and social comparison.^14^ Students working together collaboratively are thought to benefit in terms of increased self‐efficacy, motivation and by improving their metacognitive skills when processing new information.^15^, ^16^ Yet, concerns regarding procedural learning have been raised that the reduced hands‐on time associated with collaborative skills learning may affect learners' learning negatively over time, in particular if the number of collaborators increase.^15^, ^17^ The importance of extended periods of hands‐on time during clinical skills training is essential for achieving movement automaticity, which again has been shown to result in improved transfer test performances.^18^ As the number of collaborators during skills training will affect the amount of hands‐on time available per learner, transfer of learning may be equally impaired.

Although many different theories have been proposed to understand collaborative learning, little observational data have been reported in the medical education literature to provide empirical support for these explanatory frameworks. One theoretical framework that can be used to understand learning activities during collaborative learning is the ICAP framework.^12^, ^16^ This framework is based on learners' overt behaviours to reflect different modes of cognitive engagement while learning.^12^ The four behavioural modes are described as passive (P), active (A), constructive (C) and interactive (I), and each mode is associated with different examples of learning activities. The ICAP framework describes a hierarchical distribution of learning activities, where interactive activities are thought to be superior to constructive activities, which are superior to active activities, that again are superior to passive activities (I > C > A > P). As such, two potentially opposing hypotheses for collaborative skills learning have been expressed in the existing literature.

1. Collaborative learning during initial practice leads to different levels of overt learning activities in students. The higher level of cognitive engagement, the better learning outcomes.
2. Decreasing the amount of hands‐on time during practice has a negative effect on skills learning.

Yet, there is limited empirical evidence on how the number of collaborators may affect overt learning activities or skills learning. This study aimed to explore the impact of group size on ICAP activities during skills training and on subsequent transfer of skills to investigate whether increasing group size would serve as a mediator and/or a barrier to learning. The context of our study was simulation‐based ultrasound training, representing a complex procedural and diagnostic skill that is becoming increasingly relevant in several clinical specialties.

The research question was: *In a group of ultrasound novices, what are the effects of simulation‐based ultrasound training individually, in dyads, triads or tetrads with respect to learning activities during training and on subsequent skills transfer?* Gaining insight into the impact of group size on learning may help provide empirical support for existing theories that are used to understand collaborative skills learning. Moreover, exploring the impact of increasing group size on skills learning and transfer is important to how we deliver low‐cost high‐value skills training in the future.

## 2 METHODS

### 2.1 Setting

This was a randomised controlled trial using a pre‐post‐transfer‐test set‐up. The study was conducted between December 2019 and October 2020 at Copenhagen Academy for Medical Education and Simulation (CAMES), Denmark and was reported according to the CONSORT statement. The Regional Ethics Committee of the Capital Region of Denmark exempted the study from review, Protocol no: H‐19063724. The study was registered at clinicaltrials.gov (NCT04167397).

### 2.2 Participants

The participants were medical students from the University of Copenhagen. They were recruited via advertising on student fora on Facebook. The inclusion criteria were proficiency in Danish and a passed general anatomy exam. Exclusion criteria were prior ultrasound experience and any clinical experience from an OB/GYN department.

### 2.3 Randomisation

Participants were block randomised to individual, dyad, triad or tetrad training in a 1:1:1:1 allocation ratio. Randomisation was performed by an independent researcher from our institution using random permuted blocks generated online. Due to the COVID‐19 lockdown, some participants' training sessions were cancelled. The study was paused for 5 months, and data collection started again in August 2020.

### 2.4 Equipment

Pre‐ and post‐tests were completed using the transabdominal *ScanTrainer* (Medaphor, Ltd, Cardiff, UK). This simulator has a haptic device that provides realistic force feedback. During the training, participants practised the assessment of fetal growth during gestational ages 20–27 weeks. The training programme consisted of nine modules containing built‐in automated assessments with previously established validity evidence.^19^ The participants were introduced to the simulator and the tasks via video instruction to ensure consistency and received no further instruction during training. The simulator provides dichotomous feedback after the completion of each module (for example, ‘fetal abdomen correctly magnified’), which the participants were allowed to read and discuss. Technical assistance from the instructor regarding the simulator was provided if needed.

The transfer test was performed on the *U/S Mentor* simulator (3D systems health care, Littleton, USA). The transfer test simulator differs from the training simulator in terms of the quality of ultrasound images, the user interface for handling the probe and finally by reproducing a moving rather than a fixed fetus making the ultrasound scan slightly more difficult. The simulator provided no guidance regarding optimal scanning planes or help modules.

### 2.5 Interventions

**FIGURE 1:**
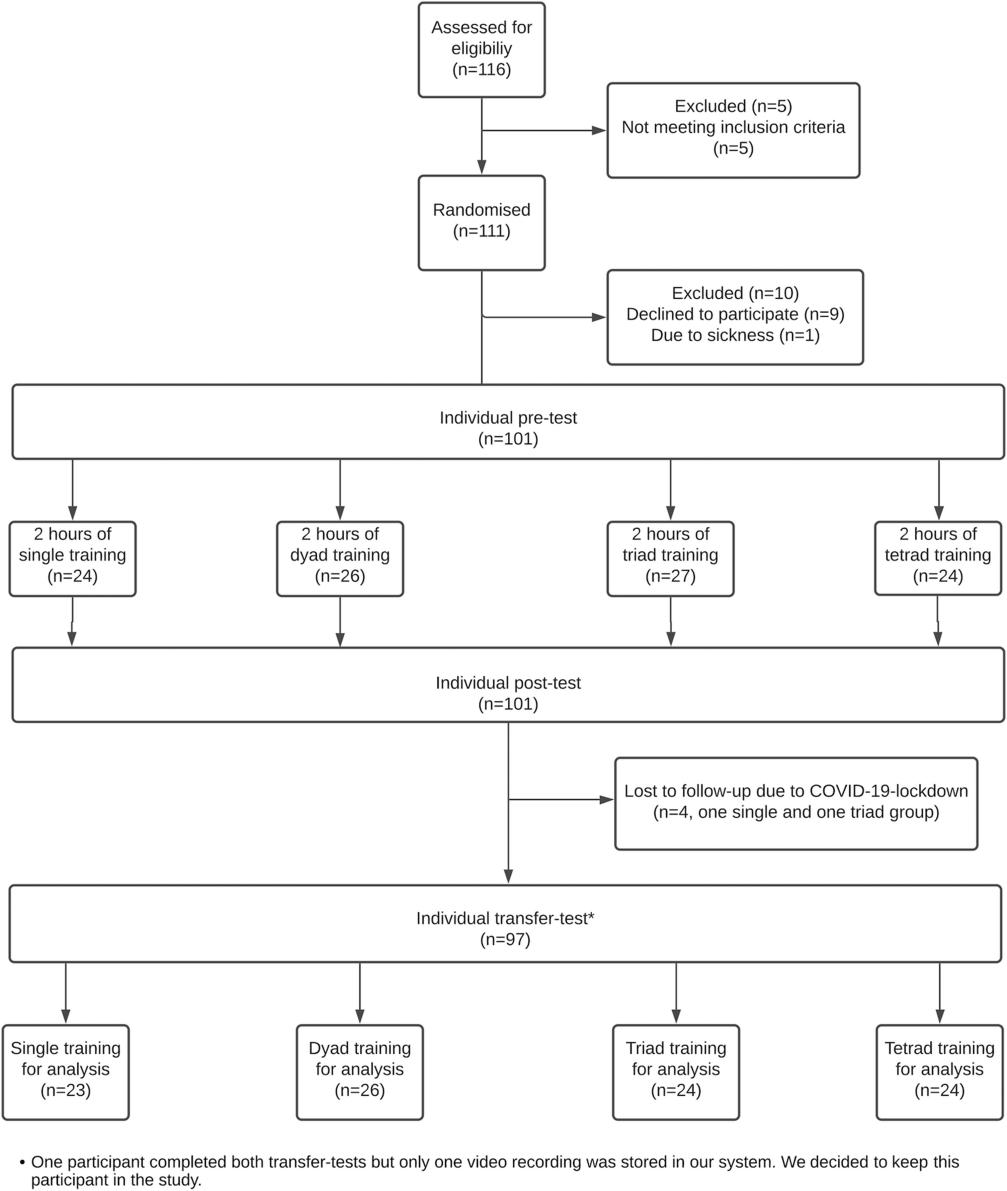
Study flowchart

The study design is illustrated in Figure 1.

The participants were randomised to either single, dyad, triad or tetrad training. They all completed an individual pre‐test, then trained in different group sizes for 2 hours and subsequently performed an individual post‐test on the Scantrainer. After 7–10 days, they completed an individual transfer test on the *U/S Mentor* simulator.

Pre‐test: The participants received a video introduction to second trimester transabdominal ultrasound and fetal biometry measurements (10 minutes) and then a video introduction to the simulator (15 minutes). Subsequently, they completed an individual pre‐test including measurements of biparietal diameter (BPD), occipito‐frontal diameter (OFD), anterior–posterior abdominal diameter (APAD), transabdominal diameter (TAD) and femur length (FL).
Training: The participants then practised the assessment of fetal biometries individually, in dyads, triads or tetrads, respectively. They trained in a fixed time frame of 2 hours, regardless of group size. The participants working in groups were instructed that they should take turns completing the assignments when training in groups, and only one person should actively handle the ultrasound probe at a time. The other participants were allowed to help with freezing images, obtaining measurements, provide suggestions, ask questions and discuss findings. The entire training session was video recorded for subsequent coding of the learning activities according to the ICAP framework.
Post‐test: Immediately after completing the 2‐hour training session, the participants underwent an individual post‐test identical to the pre‐test using the same simulator as they trained on.
Transfer test: Within the next 7–10 days, the participants performed an individual transfer test on a different simulator, the U/S Mentor simulator (Symbionix Ltd, Israel), obtaining measurements of BPD, OFD, APAD, TAD and FL in an obstetric second trimester scanning module. They performed measurements on two cases, in which the fetus was moving during the scan as well as situated in different positions (cephalic presentation/breech presentation). The participants had a maximum of 15 minutes to complete each case. The time interval between post‐test and transfer test was chosen to evaluate transfer as well as retention, while at the same time keeping participant attrition at a minimum.

### 2.6 Outcomes

The primary outcome was performance scores during the transfer test. The secondary outcomes included performance scores on the pre‐ and post‐test, ICAP distribution and hands‐on time across groups.

### 2.7 Performance scores

All test performances were video recorded directly on the simulators, where the ultrasound image, the simulator and the probe movements were visualised. Two blinded fetal medicine experts rated each performance on the Objective Structured Assessment of Ultrasound Skills (OSAUS) scale^20^ and a Global Rating Scale (GRS) (see Appendix S1). Validity evidence for the use and interpretation of OSAUS scores have been collected and demonstrated in several previous studies, including content evidence,^21^ response process, internal structure, relation to other variables and consequences.^20^, ^22^, ^23^ The OSAUS instrument includes seven items, which are evaluated on 5‐point Likert scales. The first and last items should only be rated when appropriate,^20^ and because our study took place in the simulated setting, only items 3–5 were included in the analysis, consistent with previous uses.^24^, ^25^, ^26^ Additionally, the experts also rated an overall GRS score on a 5‐point Likert scale. The GRS is based on an expert evaluation and has been used to evaluate various technical performances.^27^, ^28^ The mean of both transfer cases was used to create the primary outcome measures.

### 2.8 Rater training

All performances were rated by two blinded raters. The use of two assessors and two cases on the transfer test was chosen based on a previous study demonstrating high inter‐rater reliability (intraclass correlation coefficient [ICC] = 0.89) when using the OSAUS scale to assess fetal biometry examinations.^20^

We included a total of four raters, who were either fetal medicine consultants (LNN, LH) or had PhDs in fetal medicine (NGP and LAA). All raters were active as clinicians working with obstetric ultrasound on a daily basis. Prior to rating, all raters tried both of the simulators and the assignments the students had to complete. The four raters initially rated seven videos individually (pilot videos, not included in the analysis) and met in groups of two to discuss any inconsistencies in their ratings and watched selected sections of the videos together. The videos were then divided into four groups (A, B, C and D, 24/25 participants' videos in each group). All participants had four videos evaluated by two raters, one pre‐test, one post‐test and two transfer tests.

### 2.9 ICAP rating

LMN assessed participants' ICAP activities during training based on Chi and Wylie's framework.^12^ The ICAP coding scheme was adjusted to our local setting, e.g. by including ‘handling the simulator probe’ as an example of an active behaviour. We translated the different engagement modes into a numeric version with 0 = No learning activity, 1 = passive, 2 = active, 3 = constructive and 4 = interactive. The content of the coding scheme was discussed and revised between MGT, KK and LMN, and the final version can be seen in Appendix S2. Additionally, hands‐on time was registered separately for each individual participant. A pilot was conducted, in which ICAP ratings were co‐rated by two of the study authors (LMN and MGT). The inter‐rater agreement was sufficiently high to allow one rater to complete all assessments. All training sessions then were rated minute by minute (a total of 120 time periods per participant) and scored corresponding to highest level ICAP activity displayed for each time period.

### 2.10 Statistical analyses

We powered our study to detect large and educationally meaningful differences corresponding to a Cohen's d of 1.0 between groups. Using a power of 90% and an *α* value of 0.05, this corresponded to 22 participants in each study group. We aimed to include 25 students in each study arm to account for attrition.

The means of the two raters' scores were calculated for OSAUS and GRS scores on the pre‐ and post‐tests. A mean transfer test score from the two cases was calculated. All scores were calculated as percentages of the maximum score (maximum GRS score = 5, maximum OSAUS score = 15).

Pearson correlation coefficients were used to determine if any of the other variables including hands‐on time, pre‐test scores and post‐test scores were associated with transfer outcomes. We chose a priori to control for significant predictors of transfer performance.

The primary outcomes (mean OSAUS and GRS scores from the transfer test) were compared between the four groups using between‐subjects analysis of variance (anova), with and without adjustment for pre‐test scores as a covariate. The mean OSAUS and GRS scores from the pre‐ and post‐tests were compared between the four groups using a mixed‐design (2 × 2) repeated‐measures anova to assess the main effects of training and test for interaction with the type of training (single/dyad/triad/tetrad). Effect sizes were calculated as partial eta squared (ηp^2^). We calculated the inter‐rater reliability of the ICAP ratings using an ICC, consistency, two‐way mixed‐effects model (ICC (3,*k*)).^29^ We conducted separate generalizability analyses of GRS and OSAUS data.^30^ We specified the generalizability model with Participant nested in Group (randomised group) as the facet of differentiation. The facets of generalisation were Case (2 cases) and Rater nested in case (2 raters per case) as well as Item for the OSAUS model. We report overall reliability of the design and specific inter‐case, inter‐rater and inter‐item (for the OSAUS) generalisability coefficients. All statistical analyses were conducted using IBM SPSS Statistics 25.

## 3 RESULTS

The study included a total of 111 participants randomised to the four different training conditions; 101 participants completed the pre‐test, the intervention and the post‐test. One video recording from a triad group was not correctly saved, and the ICAP ratings were not completed for this group, and only their expert ratings were evaluated in the analysis. Four participants were lost to follow‐up due to the COVID‐19 lockdown. The participant demographics are reported in Table 1.

**TABLE 1:** Baseline demographics

Participants	Singles	Dyads	Triads	Tetrads
*n*	23	26	24	24
Age, years, mean (95% CI)	24.9 (24.3–25.6)	25.6 (24.3–27.0)	24.9 (24.1–25.7)	24.8 (23.6–26.1)
Female, *n* (%)	22 (95.7%)	26 (100%)	22 (91.7%)	20 (83.3%)
Male, *n* (%)	1 (4.3%)	0 (0%)	2 (8.3%)	4 (16.7%)
Year of medical school, *n* (%)				
Year 3	5 (21.7%)	6 (23%)	6 (25%)	6 (25%)
Year 4	6 (26.0%)	11 (42.3%)	5 (20.9%)	7 (29.2%)
Year 5	5 (21.7%)	6 (23%)	9 (37.5%)	11 (45.8%)
Year 6	7 (30.4%)	3 (11.5%)	4 (16.7%)	0 (0%)
Ultrasound experience, *n* (%)				
None	6 (26.1%)	9 (34.6%)	8 (33.3%)	8 (33.3%)
1–4 hours	17 (73.9%)	17 (65.4%)	16 (66.7%)	16 (66.7%)

### 3.1 Primary outcome

Group size did not significantly influence performance measured by OSAUS scores (F_(3,93)_ = 1.94, p = 0.13, ηp^2^ = 0.06) or GRS scores (F_(3,93)_ = 1.46, p = 0.23, ηp^2^ = 0.05) on the transfer test, Table 2. These results did not change when adjusting for pre‐test, post‐test or hands‐on time as a covariate. Hands‐on time was not correlated with transfer performance scores.

**TABLE 2:** Performance scores across the pre‐test, post‐test and transfer test

	Singles	Dyads	Triads	Tetrads	p value
OSAUS score, percentage of maximum score, Mean (95% CI)					
Pre‐test	41.0% (34.6–47.4%)	34.0% (29.4–38.5%)	37.2% (33.0–41.5%)	38.2% (33.6–42.8%)	<0.001* ^,^ ^a^
Post‐test	64.3% (56.6–72.1%)	53.7% (47.5–59.9%)	51.5% (44.9–58.2%)	57.8% (50.8–64.7%)	0.38^b^ ^,^ ^a^
Transfer test	61.5 (53.9–69.1%)	51.2% (44.7–57.7%)	57.2% (49.8–64.6%)	59.1% (54.5–63.7%)	0.13^c^
GRS score, percentage of maximum score, Mean (95% CI)					
Pre‐test	40.0% (33.7–46.3%)	34.6% (29.4–39.9%)	37.5% (31.1–43.9%)	35.4% (30.1–40.7%)	<0.001* ^,^ ^a^
Post‐test	61.3% (51.3–71.3%)	50.8% (44.7–56.8%)	51.3% (43.9–58.6%)	54.2% (46.2–62.1%)	0.59^b^ ^,^ ^a^
Transfer test	63.7 (55.4–72.0%)	53.3% (46.5–60.1%)	57.9% (49.3–66.5%)	60.0% (53.8–66.2%)	0.23^c^

*Note*: 95% CI = 95% confidence interval.

^a^ Two‐way mixed repeated measures anova comparing pre‐ and post‐test performances.

^b^ Interaction effect between group size and performance.

^c^ One‐way anova comparing transfer‐test scores.

* Significant p value.

### 3.2 Secondary outcomes

OSAUS scores improved significantly from pre‐ to post‐test for all groups as did GRS scores (OSAUS mean [SD]: 37.5% [12.0)] versus 56.7% [16.8]; F_(1,93)_ = 113.9, p < 0.001, ηp^2^ = 0.55; GRS mean [SD]: 36.8% [13.7] versus 54.2% [18.9]; F_(1,93)_ = 75.4, p < 0.001, ηp^2^ = 0.45). There was no interaction effect between group size and training (F_(3,93)_ = 1.04, p = 0.38, ηp^2^ = 0.03), Table 2.

**FIGURE 2:**
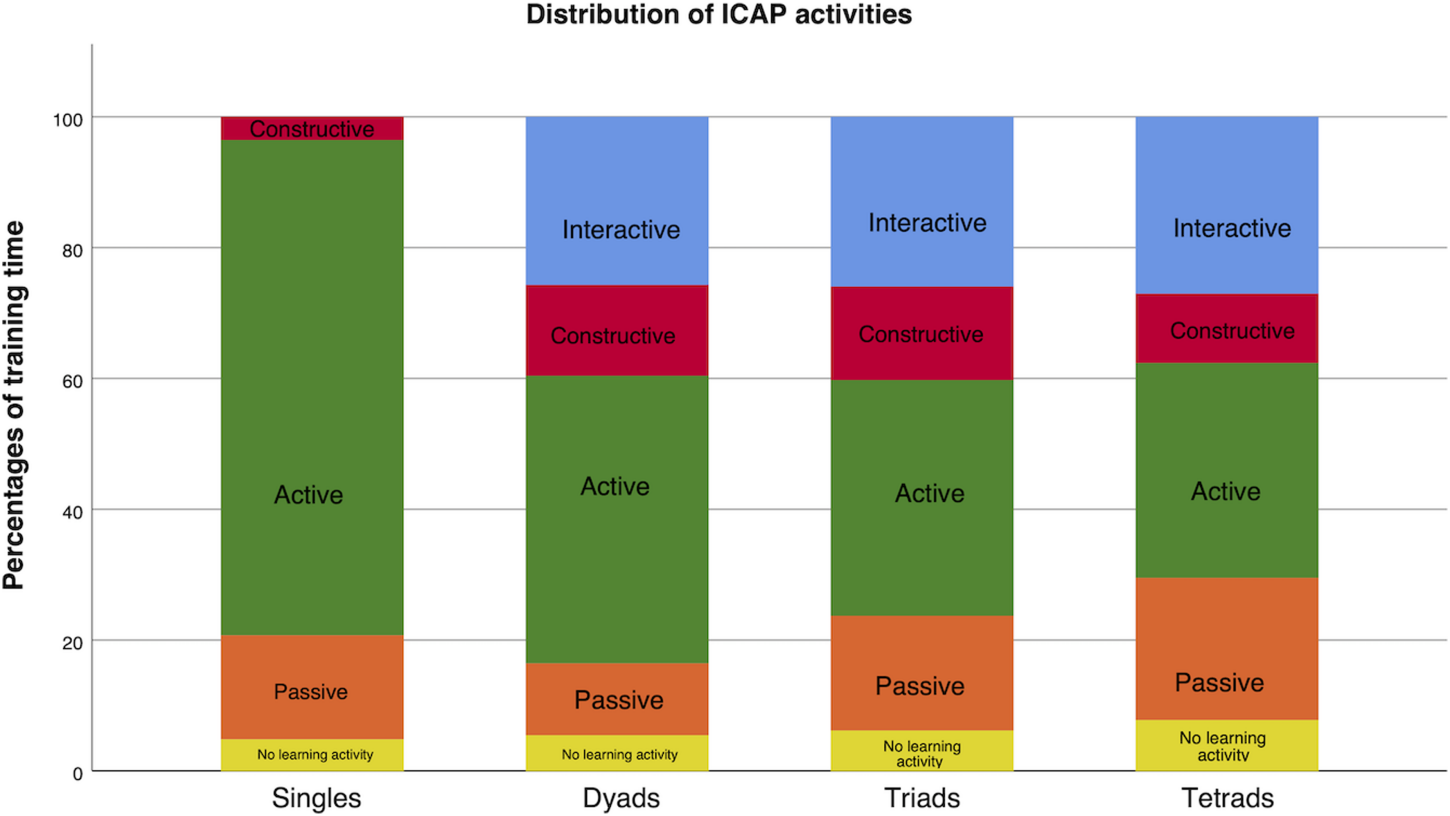
Distribution of time spent in Interactive‐Constructive‐Active‐Passive (ICAP) activities across the four groups

The distribution of time spent in each ICAP activity was significantly different between the groups for all learning activities except for the no learning activity category, Figure 2 (Passive [F_(3,90)_ = 13,75, p < 0.001, ηp^2^ = 0.31], Active [F_(3,90)_ = 139.28, p < 0.001, ηp^2^ = 0.82], Constructive [F_(3,90)_ = 10.73, p < 0.001, ηp^2^ = 0.26], Interactive [F_(3,90)_ = 43.17, p < 0.001, ηp^2^ = 0.59], No learning activity [F_(3,90)_ = 1.65, p = 0.183, ηp^2^ = 0.05]).

Time spent in the non‐learning and passive learning activities (category 0 + 1) increased with group size (mean per cent [SD] singles: 20.7 [5.2], dyads: 16.4 [8.3], triads: 23.7 [8.2], tetrads: 29.5 [7.2]; F_(3,90)_ = 13.75, p < 0.001, ηp^2^ = 0.31), Figure 2. Post hoc testing (Tukey) showed significant differences in percentage of time spent in category 0 + 1 between tetrads and all other groups (mean per cent difference [95% CI]; singles: 8.78 [3.16–14.4], p = 0.001; dyads: 13.1 [7.6–18.5], p < 0.001; triads: 5.8 [0.04–11.6], p = 0.045) and between triads and dyads (mean per cent difference [95% CI]: 7.27 [1.61–12.93], p = 0.006). Time spent in the constructive and interactive learning activities (category 3 + 4) was relatively constant between groups compared with singles (mean per cent [SD] singles: 3.5 [3.9]; dyads: 39.6 [13.3]; triads: 40.2 [9.7]; tetrads: 37.6 [8.8]; F_(3,90)_ = 78.38, p < 0.001, ηp^2^ = 0.72). Post hoc testing (Tukey) showed significant differences in percentage of time spent in category 3 + 4 (constructive and interactive) between singles and all other groups (mean per cent difference [95% CI] dyads: 36.0 [28.7–43.3], p < 0.001; triads: 36.7 [29.0–44.4], p < 0.001; tetrads: 34.1 [26.7–41.1], p < 0.001).

**FIGURE 3:**
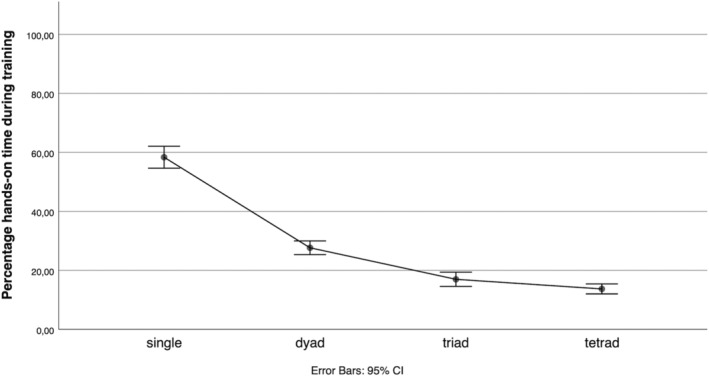
Percentage hands‐on time spent by each individual across the four groups

The amount of hands‐on time (percentage of the training time each participant was handling the ultrasound probe) decreased with increasing group size (mean per cent [SD]: singles: 58.4 [8.6], dyads: 27.7 [5.7], triads: 17.0 [5.3]; tetrads: 13.7 [4.0]; F_(3,90)_ = 252.54, p < 0.001, ηp2 = 0.89). Post hoc testing (Tukey) revealed that this difference was statistically significant between singles and all other groups (mean per cent difference [95% CI] dyads: 30.7 [26.1–35.3], p < 0.001; triads: 41.4 [36.5–46.2], p < 0.001; tetrads: 44.6 [40.0–49.3], p < 0.001), between dyads and larger groups (mean per cent difference [95% CI] triads: 10.7 [6.0–15.4], p < 0.001; tetrads: 13.9 [9.4–18.5], p < 0.001) but not between triads and tetrads (mean per cent difference [95% CI]: 3.3 [−1.5 to 8.1], p = 0.29), Figures 2 and 3. Group size and hands‐on time were negatively correlated (Pearson's r = −0.86, p < 0.001).

The ICC (3,*k*) for the ICAP ratings was 0.70 (95% CI: 0.60–0.77). The generalisability theory analysis of our data showed that inter‐case generalisability coefficients for the GRS and OSAUS scores were high at 0.84 and 0.88, respectively. Inter‐rater generalizability coefficient within case was 0.72 and 0.81, respectively. Inter‐item generalisability coefficient within a single case and rater for the OSAUS scores was 0.75. The overall reliability of the design for the GRS scores was 0.64 and 0.60 for the OSAUS scores.

## 4 DISCUSSION

Collaborative skills training in group sizes of up to four participants did not negatively impact skills transfer despite less hands‐on time. All participants improved about 15–20 percentage points with training irrespective of group size. This is to some extent surprising because some participants had as little as 25% of the hands‐on time of those who trained alone. These findings align with and expand on previous studies involving collaborative learning in pairs (dyad learning) by demonstrating that the positive effects of collaborative skills learning also expand to larger groups of learners.^4^, ^8^, ^9^, ^10^, ^31^ Currently, there is an increased focus on collaborative and peer learning strategies in medical education, including how these strategies may meet the demand for high‐value low‐cost education.^15^, ^32^ Simulation‐based training requires a significant amount of resources (e.g. instructor and equipment costs among others), and our findings may help reduce these demands by decreasing the simulator: learner ratio without any negative effects on learning outcomes.

We suggest that there may be a trade‐off between decreasing hands‐on time and increasing time spent on observation of and interactions with other learners. In our study, the time spent in constructive and interactive activities did not differ between dyads, triads and tetrads, and even though participants in dyads had more hands‐on time than participants in triads or tetrads, they still had similar learning outcomes. These findings indicate that participants seemed to compensate for the reduced hands‐on time through higher levels of cognitive engagement as a consequence of collaborative efforts. Specifically, during training of procedural skills such as ultrasound, collaborative learning can prove beneficial, as there are multiple tasks that should be performed simultaneously (e.g. manipulating the probe, adjusting machine settings and communicating with the patient while engaged in the visual diagnostic task). Distributing tasks between learners is a potentially beneficial strategy as it allows learners to stay engaged in learning without cognitive overload.^5^ Other advantages may include assisting visual search and discussing anatomical features or pathology that normally require a second look by an experienced clinician.

We know from previous studies that the duration of our intervention (2 hours) was sufficient to induce an effect but less than required for the learning curves to flatten (+4 hours) leading to a ceiling effect.^19^ Time‐on‐task and thereby the amount of hands‐on time available are normally the most important determinants of learning; however, in our study, observation and higher‐order ICAP activities during collaborative learning seemed to outweigh the reduced hands‐on time. Yet, because hands‐on time and ICAP activities were both confounded by group size, we cannot infer directly the extent to which these two opposing forces contributed to the learning outcomes observed in our study.

A recent study by Shebilske et al. demonstrated similar learning outcomes between individuals, dyads, triads and tetrads when learning a video task for pilot training.^33^ The authors primarily ascribed their results to the positive effects of observation.^34^ Observational learning is (among other theories) supported by the mirror neuron hypothesis, which claims that areas in the premotor cortex are activated both during the performance and observation of a skill.^5^, ^31^, ^33^, ^35^ Our results support observation as a key reason for similar transfer results between groups but expands this finding by suggesting that other social and interactive mechanisms are involved in collaborative complex skills learning, in particular for practical skills learning.^15^ While the traditional ICAP framework places observation as a passive activity,^12^ it is inseparable from the higher‐order learning activities because it serves as a basis on which constructive and interactive activities unfold during clinical skills training. However, observation is categorised as a passive activity regardless of the cognitive level of engagement, which may not be compared with passiveness as a result of fatigue, lack of motivation or concentration or from boredom. Although the ICAP framework is supposed to cover all kinds of learning activities, it may undervalue the role of observation for learning practical skills. In this sense, the concept of observation may need to be expanded to include various degrees of cognitive engagement to fully understand its role in clinical skills learning and to better conceptualise activities during practical skills learning.

We found that participants in triads and tetrads spent more time in the non‐learning and passive learning categories (0 + 1) than the participants in dyads and single groups. This could be due to increased commitment and intimacy between learners in the dyad constellations, which may decrease with increasing group size.^36^ We (incorrectly) anticipated that the increase in non‐learning and passive activities would have led to lower levels of skills transfer in the large groups. Although this was not the case, we expect that the balance between the amount of hands‐on time provided and collaborative compensatory effects must at some point tip for larger groups (more than four learners). Exploring under which conditions participants remain motivated and consider it meaningful to collaborate is an important aspect to consider when dealing with larger group sizes as this has been a cause of concern voiced by learners in previous studies.^37^, ^38^, ^39^

### 4.1 Strengths/limitations

Strengths of this study include the randomised design, the large sample size included, the use of assessment scores with established validity evidence and the use of transfer tests to reflect the key outcome of interest.

The study also has some limitations. We focused on novices and on a single complex clinical procedure. Future research needs to establish whether our findings translate to more advanced learners and to different types of practical skills (e.g. simple versus complex skills or diagnostic versus therapeutic procedures). Most of the participants were women; however, we have chosen not to focus on gender in this study as the participants were randomised and subsequently performed equally on their pre‐test. Therefore, we did not find it relevant to discuss the influence of gender any further in this context. The students trained with feedback only generated by the simulator. Uninstructed practice may lead to misunderstandings of the assignments without supervision by an instructor, which may have provided an uneven advantage for the larger groups to correct errors and misunderstandings. We only explored group sizes of up to four participants as we expected four participants to be the upper limit to remain motivated. While we did observe an increase in passive and non‐learning activities with increasing group size, we did not identify an optimum group size or a point, in which learning outcomes worsened. To do this, we would have had to either include several larger groups or increase our sample size 10‐fold to demonstrate small differences in transfer outcomes, both of which would have been infeasible. Finally, there may have been differences in transfer‐test performances that were below the magnitude we powered our study to detect. The question is, however, if small differences are of relevance within the field of simulation‐based medical education, where gains are usually large.

## 5 CONCLUSION

Collaborative skills learning in group sizes of up to four participants did not impair skills transfer despite less hands‐on time. This finding may be explained by the role of observation combined with a compensatory shift towards constructive and interactive learning activities that outweigh the negative effects of diminishing hands‐on time.

## CONFLICT OF INTEREST

The authors declare that they have no conflicts of interest.

## AUTHOR CONTRIBUTIONS

LMN was responsible for writing the study protocol, the data collection and data analysis and is the first author of the paper. AMM and KK were responsible for co‐writing the study protocol and data analysis and are co‐writers of the paper. LNN, LH, NGP and LAA were responsible for performance ratings and are co‐writers of the paper. VJ and AV were co‐responsible for data collection and are co‐writers of the paper. MGT was responsible for planning the study, co‐writing the study protocol, and data analysis and is co‐writer of the paper.

## ETHICS STATEMENT

The Regional Ethics Committee of the Capital Region of Denmark exempted the study from review, Protocol no: H‐19063724.

## Supporting information




**Appendix** S1. OSAUS and GRS scores

**Appendix** S2. ICAP coding scheme.
