# Supplementary material for: Does group size matter during collaborative skills learning? A randomised study
Source: Med Educ. 2022 Mar 16;56(6):680–9. doi: 10.1111/medu.14791 (PMC9313549; doi:10.1111/medu.14791)
Supplement: Supplementary file 1 — Appendix S1. OSAUS and GRS scores [file MEDU-56-680-s002.pdf]

Appendix A

The OSAUS Scale:

**1. Indication for the examination**	1	2	3	4	5
If applicable. Reviewing patient history and knowing why the examination is indicated.	Displays poor knowledge of the indication for the examination		Displays some knowledge of the indication for the examination		Displays ample knowledge of the indication for the examination
**2. Applied knowledge of ultrasound equipment**	1	2	3	4	5
Familiarity with the equipment and its functions, i.e. selecting probe, using buttons and application of gel.	Unable to operate equipment		Operates the equipment with some experience		Familiar with operating the equipment
**3. Image optimization**	1	2	3	4	5
Consistently ensuring optimal image quality by adjusting gain, depth, focus, frequency etc.	Fails to optimize images		Competent image optimization but not done consistently		Consistent optimization of images
**4. Systematic examination**	1	2	3	4	5
Consistently displaying systematic approach to the examination and presentation of relevant structures according to guidelines.	Unsystematic approach		Displays some systematic approach		Consistently displays systematic approach
**5. Interpretation of images**	1	2	3	4	5
Recognition of image pattern and interpretation of findings.	Unable to interpret any findings		Does not consistently interpret findings correctly		Consistently interprets findings correctly
**6. Documentation of examination**	1	2	3	4	5
Image recording and focused verbal/written documentation.	Does not document any images		Documents most relevant images		Consistently documents relevant images
**7. Medical decision making**	1	2	3	4	5
If applicable. Ability to integrate scan results into the care of the patient and medical decision making.	Unable to integrate findings into medical decision making		Able to integrate findings into a clinical context		Consistent integration of findings into medical decision making

doi:10.1371/journal.pone.0057687.t003

The Global Rating Scale:

On a Likert scale from 1 – 5, please identify the full performance according to what can be expected of a medical student who has completed two hours of training in obstetric ultrasound and otherwise has no OBGYN ultrasound experience.

1:	2	3:	4	5:
Poor performance		Standard performance		Excellent performance
