# Supplementary material for: Does group size matter during collaborative skills learning? A randomised study
Source: Med Educ. 2022 Mar 16;56(6):680–9. doi: 10.1111/medu.14791 (PMC9313549; doi:10.1111/medu.14791)
Supplement: Supplementary file 2 — Appendix S2. ICAP coding scheme. [file MEDU-56-680-s001.pdf]

Appendix B

Definition	Non-verbal behavior	Verbal behavior
P = Passive = 1<br>*RECEIVING*<br><br>*"Learners being oriented toward and receiving information from the instructional material without overtly doing anything else in relation to learning."*	• Reading a text silently<br>• Watching a video<br>• Looking at the posters with pictures of the 3 scanning planes (both handouts and taped to the wall of the room)<br>• Reading feedback on the screen and touching the screen to scroll up and down.<br>• Handling shifting between the assignments.	• Silence<br>• Listens and utters agreement ("mmm, uh huh…")
A = Active = 2<br>*MANIPULATING*<br><br>*"Some form of overt motoric action or physical manipulation is undertaken."*	• Pointing or gesturing towards the screen<br>• Looking at the posters with pictures of the three scanning planes AND gesturing, pointing, repeating words, etc. (both handouts and taped to the wall of the room)<br>• Handling the probe during the assignments.<br>• Handling the touch screen during the assignments.	• Repeating what was already said in the instructional material (content from the videos, the simulator, the posters)<br>• Reading text aloud<br>• Yes/No answers<br>• Taking notes<br>• Repeating what another participant said without elaborating
C = Constructive = 3<br>*GENERATING*<br><br>*"Learners generate or produce additional externalized outputs or products beyond what was provided in the learning materials"*	• Drawing a concept map<br>• Self-evalutating or monitoring one's performance<br>• Taking notes in one's own words<br>• Looking things up online on a cell phone (Cannot be coded unless they actively say that that's what they're doing or when we can see the content on the screen, for example ultrasound images)<br>• Drawing a concept map	• Explaining to others or oneself<br>• Reflecting out loud<br>• Inducing hypotheses<br>• Asking questions<br>• Comparing and contrasting cases<br>• Two (or more) people can be constructive without interaction, then it's two (or more) individual dialogues.

I = Interactive = 4<br>*DIALOGUING*<br><br>*"Dialogues that meet two criteria:*<br>*1. Both (all) partners' utterances must be primarily constructive*<br>*2. a sufficient degree of turn taking must occur."*	• Two or more partners are collaborating (i.e. one is handling the probe, another one is handling the touch screen, etc.)	• Two or more partners are discussing a problem (co-construction of knowledge)<br>• All partners' utterances must be primarily constructive<br>• Defending or arguing a position<br>• Asking and answering comprehension questions with a partner(s)<br>• Elaborating on each other's contribution<br>• Relatively frequent turn taking<br>• Drawing a map together with one or more partners
N = No learning activity = 0	• Leaving the room<br>• Looking around in the room/walking around in the room without paying attention to the simulator.<br>• Looking at a cellphone (and not paying attention to the simulator as well)<br>• Eating<br>• Handling technical difficulties	• Private telephone calls<br>• Private talk with instructor<br>• Private conversation with group member(s)<br>• Talking about technical difficulties<br>• Talking about how to log into the assignments, if it's the correct one, etc.
